# The shift of microbial communities and their roles in sulfur and iron cycling in a copper ore bioleaching system

**DOI:** 10.1038/srep34744

**Published:** 2016-10-04

**Authors:** Jiaojiao Niu, Jie Deng, Yunhua Xiao, Zhili He, Xian Zhang, J. D. Van Nostrand, Yili Liang, Ye Deng, Xueduan Liu, Huaqun Yin

**Affiliations:** 1 School of Minerals Processing and Bioengineering, Central South University, Changsha 410083, China; 2 Key laboratory of Biometallurgy, Ministry of Education, Changsha 410083, China; 3 Institute for Environmental Genomics and Department of Botany and Microbiology, University of Oklahoma, Norman 73019, USA; 4 Research Center for Eco-Environmental Sciences, Chinese Academy of Sciences, Beijing 100081, China

## Abstract

Bioleaching has been employed commercially to recover metals from low grade ores, but the production efficiency remains to be improved due to limited understanding of the system. This study examined the shift of microbial communities and S&Fe cycling in three subsystems within a copper ore bioleaching system: leaching heap (LH), leaching solution (LS) and sediment under LS. Results showed that both LH and LS had higher relative abundance of S and Fe oxidizing bacteria, while S and Fe reducing bacteria were more abundant in the Sediment. GeoChip analysis showed a stronger functional potential for S^0^ oxidation in LH microbial communities. These findings were consistent with measured oxidation activities to S^0^ and Fe^2+^, which were highest by microbial communities from LH, lower by those from LS and lowest form Sediment. Moreover, phylogenetic molecular ecological network analysis indicated that these differences might be related to interactions among microbial taxa. Last but not the least, a conceptual model was proposed, linking the S&Fe cycling with responsible microbial populations in the bioleaching systems. Collectively, this study revealed the microbial community and functional structures in all three subsystems of the copper ore, and advanced a holistic understanding of the whole bioleaching system.

In recent years, bioleaching has been widely applied on an industrial scale due to the advantages of low cost, being environment friendly, and its potential in treatment of complex and low-grade ores^1^. Microorganisms play important roles in the process, and studies of microbial community composition, structure and function in related systems have been conducted^2^,^3^,^4^. As the environmental equivalent of bioleaching system, acid mine drainage (AMD) have been comprehensively researched and shown to be mainly composed of *Nitrospira*, *Alphaproteobacteria*, *Gammaproteobacteria*, *Deltaproteobacteria*, *Acidobacteria*, *Actinobacteria* and *Euryarchaeota*^4^,^5^,^6^. A considerable fraction of Fe and/or S oxidizers were ubiquitously detected in AMD related environments, and proven to be important for the ecosystem function^7^. The associated functional genes/enzymes have also been assayed, such as sulfur oxidation multienzyme complex (*Sox*), dissimilatory sulfite reductase (*DsrAB*) and rusticyanin^8^,^9^. However, the complete bioleaching process is complex and may involve microbial communities with different compositions and functions. During bioleaching, solid compounds in leaching heap (LH) are transformed (by acid and microbes) into soluble and extractable elements, which are washed into a collection reservoir, forming leaching solution (LS). And leaching solution is pumped and sprinkled on the leaching heap periodically. The LS is an extreme environment, with low pH and high concentrations of metals, while the sediment under the LS is anaerobic or micro-aerobic^10^. LH, LS and Sediment compose three key subsystems during bioleaching^2^,^11^, but how microbial communities shift in composition, structure and function among the three subsystems have not been studied.

Iron and sulfur are major energy sources for microorganisms in copper ore bioleaching system, and bioleaching process always companied with transformation of Fe&S compounds. The metabolism and cycling of Fe and S have been identified in many environments, such as mine tailings, AMD and acidic river^7^,^10^,^12^,^13^, which are comparable to subsystems in bioleaching system. Korehi *et al*. profiled the microbial diversity in a marine shore sulfidic mine tailings dump, similar to the LH in bioleaching system. And a model was constructed to reveal the relationship between pyrite oxidation and microbial communities^14^. Another study modeled how microbial populations participated into Fe and S cycles in Tinto River^15^, which was an acidic aquatic environment comparable to the LS subsystem. However, a complete bioleaching system is composed of three subsystems, and all the microbial communities of three subsystems might participate in mineral dissolution and Fe/S cycling. In order to reveal the whole bioleaching process, microbial components and functional potentials in all three subsystems were investigated in this study. Revealing Fe and S cycling in bioleaching system may provide a new perspective to understand bioleaching mechanism and biogeochemical Fe and S cycling.

The bioleaching system in Dexing Copper Mine (Jiangxi, China) has been observed and studied for more than 30 years, providing several major advantages for this study: (i) We have relatively complete background knowledge about this environment and inhabiting microorganisms^3^,^16^; (ii) The site is a stable system with a known cycle period (eight months); (iii) It is also a typical system with the three key nodes (LH, LS and Sediment) closely linked together^17^. In this study, we analyzed the microbial community composition from all three bioleaching subsystems using Illumina sequencing of 16S rRNA gene amplicons, the functional structures using functional gene arrays, and assessed their associations with bioleaching performance and environmental factors. Here we hypothesize that: (i) microbial community composition, function and co-occurrence patterns are different among LH, LS and Sediment and (ii) these changes of microbial communities are associated with S and Fe cycling processes. Understanding the microbial components and functional potentials in this system will ultimately benefit improve of the efficiency and rates of the leaching system.

## Results

### Physicochemical characteristics of samples

All samples recorded low pH in the range of 2.4–3.2 and high concentrations of Fe, S and Cu (Table 1). The concentrations of Fe, S and Cu in LS was 500, 8227 and 171 (ppm.) respectively. The contents of dissolved oxygen (DO) was 0.73, 1.33 and 0.39 ppm. for LH, LS and Sediment respectively. The concentrations of Fe^3+^ (36.8, 362.3, 90.7 ppm.) was higher than Fe^2+^ (32.1, 98.7, 56.9 ppm) for samples in all three subsystems, and there was more iron, including total Fe, Fe^2+^ and Fe^3+^, in Sediment than LH. The chromium reducible sulfur (CRS) content was higher than elemental sulfur (ES), and ES was higher than acid volatile sulfide (AVS) for LH and Sediment, but there was more AVS in LS. Comparing the sulfur content between LH and Sediment, CRS and ES were more abundant in LH than Sediment while AVS was more abundant in Sediment. For LS, however, the amount of CRS was lower than AVS and higher than ES. The content of total organic carbon (TOC) was 1243.2, 11.1, 8362.4 ppm, and total organic nitrogen (TON) was 263.1, 54.1, 1137.3 ppm for LH, LS and Sediment respectively.

### Phylogenetic/taxonomical profiling of microbial communities

After resampling, we kept 20000 high-quality 16S rRNA gene sequences per sample, which were classified into 1,221 OTUs. The rarefaction curves showed that our sequencing efforts were sufficient for this study, and the detected OTUs accounted for 86.47% to 89.56% of predicted OTUs (Figure S1). Both Shannon diversity (3.01 for LH, 1.49 for LS and 2.74 for Sediment) and Pielou evenness indices (0.56, 0.28 and 0.57, respectively) were low in all three subsystems, but they were significantly lower (p < 0.05) in LS than those in LH and Sediment (Table S1).

**Figure 1:**
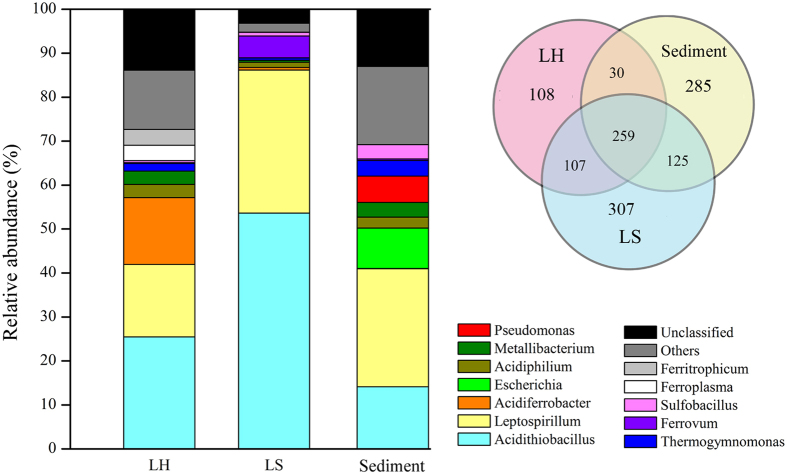
Microbial community composition and structure of each subsystem and Venn diagram depicts the shared and distinct OTUs of three environments.

Microbial community composition was significantly different in the three subsystems, confirmed by dissimilarity tests (pairwise MRPP *p* < 0.001). Detrended correspondence analysis (DCA) showed separation of LH from LS and Sediment samples, and partial overlap by LS and Sediment samples (Figure S2). Venn diagram showed that 259 OTUs were shared by all three groups. For OTUs shared by two groups, LS shared 107 and 125 OTUs with LH and Sediment respectively, while only 30 OTUs were shared by LH and Sediment. Microbial communities in all three subsystems were dominated by *Acidithiobacillus* (25.42%, 53.64%, 14.11% for LH, LS, Sediment respectively) and *Leptospirillum* (16.54%, 32.51%, 26.85%), with the next most abundant genera varying across sites: *Ferroplasma* (3.53%), *Acidiphilium* (3.02%) and *Thiomonas* (2.80%) in LH; *Acidiphilium* (1.21%) and *Sulfobacillus* (0.83%) in LS; *Escherichia* (9.20%), *Pseudomonas* (6.01%) and *Thermogymnomonas* (3.57) in Sediment (Fig. 1). For the dominant genera, *Acidiferrobacter* was more abundant in LH; *Acidithiobacillus* and *Ferrovum* were more abundant in LS; whereas Sediment samples had higher abundance of *Escherichia* (Figure S3).

**Figure 2:**
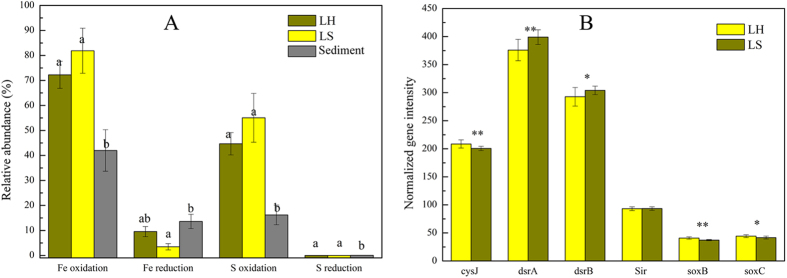
(**A**) Relative abundances of functional bacteria in three groups; (**B**) Normalized intensity of genes involved in sulfur metabolism. Significant (p < 0.05) differences among groups are indicated by alphabetic letters or *. (*p < 0.1, **p < 0.05).

In order to evaluate functional potentials of the microbial communities, we summarized all the known Fe&S metabolizers detected in this study (Table S2), and the relative abundances of microbial genera with the same function (oxidize Fe/S or reduce Fe/S) were added. For example, the relative abundances of microbial genera capable of oxidizing Fe were added to represent the abundance of Fe oxidizers in each subsystem. Results showed that microbial populations known to be capable of sulfur oxidation together accounted for 16.19% to 55.04% of total abundances, and were significantly (p < 0.05) more abundant in LH and LS than in Sediment (Fig. 2A). Similarly, relative abundances of OTUs related to known Fe oxidizers (42.00%, 72.26%, 81.89%) were significantly (p < 0.05) higher in LH and LS than in Sediment. Fe reducers were more abundant in Sediment (13.59%) than LS (3.46%), and S reducers (0.01%, 0.01%, 0.07%) were more abundant in Sediment than LH and LS.

### Microbial community functions shift across the three subsystems

S and Fe oxidation activity of microbial communities was measured during shake-flask culture, and results showed that S oxidation activity was the highest in LH and lowest in Sediment, and higher Fe oxidation activity was observed in LH and LS than in Sediment. The concentrations of Fe^2+^ in LH samples decreased rapidly after 24 hours’ incubation, but in LS and Sediment samples, decreasing started after 48 hours. After 96 hours’ incubation, the Fe^2+^ levels in LS were comparable to those in LH while the Sediment samples still lagged behind. Similarly, pH of the culture solution decreased fastest in LH and slowest in Sediment (Figure S4).

As microbial communities from LH and LS showed different Fe and S oxidation activities which could not be explained by their composition, GeoChip 5.0 was used to investigate their differences in functional potentials. A total of 28,992 gene variants were detected, including genes associated with C, N, P and S cycling, metal homeostasis, organic remediation and secondary metabolism. Notably, many genes involved in S metabolism were identified, such as sulfur oxidation (*soxABCYV*), sulfide oxidation (*sqr, fccab*) and sulfite reduction (*sir, dsrAB*).

Gene arrays revealed significant differences in functional gene structures of microbial communities from LH and LS (Table S3) across a number of ecologically important functional gene categories. The intensity of genes involved in carbon fixation was higher in LH, while genes in carbon degradation were more abundant in LS; the intensity of genes involved in nitrification was significantly higher in LS, while denitrification in LH; LS had more polyphosphate synthesis genes while LH had more polyphosphate degradation genes. The results suggested C, N and P cycles between two subsystems. Although the total intensity of genes related to S cycling showed almost no difference between two subsystems, the abundances of genes involved in sulfur oxidation (*soxB* and *soxC*) was significantly (p < 0.05) higher in LH than in LS, while *dsrA* and *dsrB* genes encoding dissimilatory sulfite reductase were more abundant in LS. A sulfur reduction gene (*cysJ*) was found to be more abundant in LH than LS (Fig. 2B). The results showed LH and LS microbial communities have different functional potentials related to bioleaching.

### Co-occurrence networks of microbial communities in each subsystem

To discern the phylogenetic molecular ecological networks of the microbial communities in the three subsystems, 16S rRNA gene amplicon sequence data were analyzed using a RMT-based network approach^18^. Three co-occurrence pMENs (LHN, LSN and SN for LH, LS and Sediment respectively) were constructed with the same threshold (0.80), in which nodes represent OTUs and links represent possible correlations among these OTUs. Major topological properties of those three networks showed that there were more nodes and links in LHN (154 nodes, 753 links), fewer in LSN (90 nodes, 378 links), and fewest in SN (53 nodes, 114 links) (Table S4). These networks also differed substantially from each other in average clustering coefficient, average path distance and modularity. The cooperation between Fe oxidizers and S oxidizers was suggested in ecological networks, as most of the links between them (79% to 100%) were positive. We further analyzed the network interactions of *Acidithiobacillus*, the major genus in all three groups, which also had the greatest number of connections. OTUs representing Fe or S oxidizing microbes only accounted for 50% of all nodes in LHN, the rest of which involved many heterotrophic and unclassified OTUs (Figure S5). For example, two OTUs of *Dyella* were showed in network and positively linked with *Thiomonas*.

### The impact of geochemical properties on microbial communities

Mantel test of taxa abundances at the genus level and environmental attributes were conducted to explore the impact of geochemical properties on microbial community composition. Because oxygen affects the valence state and content of Fe and S compounds, we controlled the effect from DO when analyzing the impacts of AVS, CRS, ES, Fe^2+^ and Fe^3+^ on microbial communities. Generally, pH, DO and the concentrations of energy sources were correlated with relative abundances of a number of microbial genera (Table S5). For example, the concentration of Fe^2+^ was significantly (r = 0.183, p = 0.039) correlated with *Acidithibacillus*, and ES had a significant impact on the distribution of *Thiobacillus*, *Thiomonas* and *Desulfitobacterium*. In addition, mantel test of functional gene structure showed that the amounts of total sulfur and reduced inorganic sulfur (RIS), including AVS, CRS and ES, significantly (p < 0.05) influenced S metabolism genes, including *sox*, *dsr*, *sqr* and *sir* (Table S6).

## Discussion

Bioleaching has been employed commercially to recover precious metals (e.g., copper, gold) from low grade ores, but one obvious shortcoming is overlong production cycle^2^. Therefore, to enhance the production efficiency becomes one of the major goals of biohydrometallurgy research. However, previous studies only focused on one of the three subsystems of bioleaching, lacking a systematic insight. Revealing the geochemical conditions as well as microbial communities change across the three subsystems would provide a holistic view of the bioleaching mechanism, and ultimately benefit achieving the goal of improving leaching efficiency.

The major finding of this study was that microbial communities of three subsystems had different Fe/S oxidation activity. It is commonly acknowledged that the ecological function of microbial communities is largely depended on their composition and structure^19^,^20^. The relative abundances of OTUs related to S and Fe oxidation were higher in LH and LS than in Sediment, resulting in higher S and Fe oxidation activity in LH and LS. Previous studies supported that the oxidation of sulfur and iron usually took place in mine tailings or acid mine drainage, and Fe/S oxidizers were frequently detected in these environments, such as *Acidithiobacillus* and *Leptospirillum*^2^,^5^,^14^. OTUs capable of Fe or S reduction were more abundant in Sediment. Oxygen content is usually a key factor determining the abundances and ecological niches of acidophiles^21^,^22^. In Sediment, relatively low oxygen levels resulted in greater abundances of anaerobic or facultative anaerobic bacteria, such as *Metallibacterium*, *Acidiphilium*, and *Desulfitobacterium*. Therefore, though not characterized, Sediment microbial communities might have stronger S and Fe reduction potentials, which requires further experimental validation.

Sulfur oxidation activity was higher by microbial communities from LH than those from LS, coincident with observations based on GeoChip analysis, that the intensity of genes involved in sulfur oxidation (*soxB* and *soxC*) was higher in LH than LS. It has been proved that AMD microorganisms adapted to the different environmental conditions via regulating the functional composition and expression of genes^23^. Here, the availability of energy resource was a key factor determining the difference of LH and LS microorganisms in sulfur oxidation. Most of RIS were insoluble in LS, including AVS (FeS, Fe_3_S_4_), CRS (FeS_2_) and S^0^. We detected lower levels of S^0^ (0~22.1 mg/L) in LS samples than LH, which explained lower elemental sulfur oxidation activity of LS microorganisms. The result was also supported by a previous study which indicated that the main role of attached acidophilic bacteria was to oxidize elemental sulfur and dissolution of chalcopyrite^24^. Interactions among microorganisms might be another reason resulted in the different Fe/S oxidation activity of three groups of microbial communities. By phylogenetic molecular ecological network analysis, we found that LHN had the largest number of nodes and links while SN had the fewest, suggesting there were strongest interactions among microbial populations in LH. A few studies suggest certain environmental conditions may result in tighter connected networks^25^,^26^, but the relationship between co-occurrence pattern of a community and its ecological roles have been rarely assessed^27^. Major dissolution reactions took place on the surface of ores in the leaching heap^28^, in this study microbial communities of LH showed highest S and Fe oxidizing activities, which might resulted from tight connection among microorganisms. It was reported that the co-culture of *Acidithiobacillus ferrooxidans* and *Acidiphilium acidophilum* enhanced their growth and iron oxidation activity^29^, supporting our speculation.

**Figure 3:**
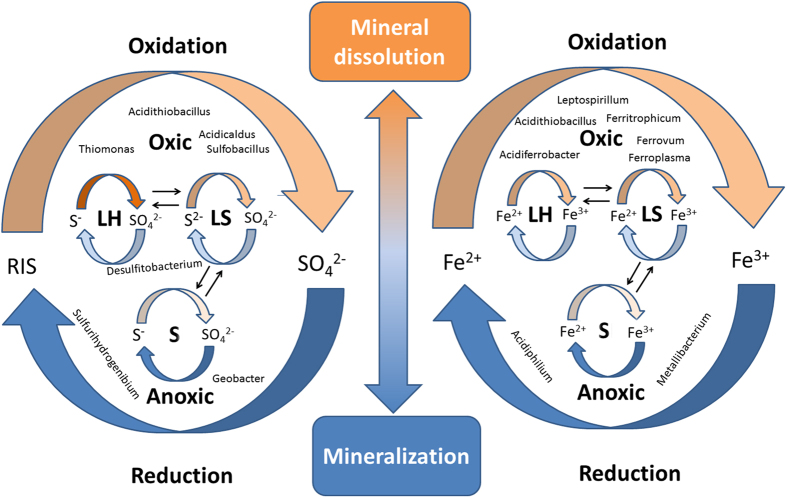
Concept model of biogeochemical Fe&S cycling and microorganisms involved in them in bioleaching system. Orange arrows represent oxidation reaction and blue arrows indicate reduction reaction. Deeper color indicates more active reaction. For example, there is stronger S oxidation in LH and stronger S reduction in Sediment. Microbial genera name is labeled at the correspondent geochemical process that the population involved.

Spatial relationship of three subsystems also influenced their microbial composition and function. Because leaching solution was pumped and sprinkled on the LH periodically, LS shared a large percent of OTUs with LH and Sediment respectively, but relatively few OTUs were shared by LH and Sediment. However, previous studies about microbial ecology of mine environments only focused on the relationship between microbial communities and physical-chemical conditions of the environment^4^,^30^,^31^, so the biochemical Fe&S cycling was modeled only within the environment^6^,^32^. Unlike many abandoned mining sites or naturally formed AMD or acidic river, Dexing bioleaching system is still in industrial application and under artificial management, thus iron and sulfur cycles among three environments (Fig. 3). Take sulfur cycling as an example: metal sulfides (chalcopyrite and pyrite) in LH were oxidized by Fe^3+^, oxygen, or microorganisms (mainly *Acidithiobacillus* and *Thiomonas*), generating RIS, including thiosulfate and elemental sulfur^33^,^34^. Then sulfur-oxidizing bacteria would oxidize RIS to generate sulfuric acid, which may accelerate mineral dissolution through the function of several detected genes (*SoxABCVY*, *Sqr*, *Fccab*). Later, all of these S compounds, including RIS and sulfuric acid, were washed into the LS, where RIS continued to be oxidized into sulfate^33^,^34^. Then a small part of RIS and most of the sulfate precipitated into the sediment, where anaerobic bacteria (e.g., *Geobacter* and *Desulfitobacterium*) reduced sulfate into S^0^ or HS^−^ through functional genes like *dsrA* and *dsrB*. The reduced inorganic sulfur may be utilized by microorganisms in LS or Sediment, or be pumped back to LH and oxidized again, thereby closing the S cycle. In previous studies, *Acidithiobacillus* was reported as the major sulfur oxidizer both in abandoned Mynydd Parys mine and Tinto River^15^,^32^, but different valence state of sulfur and sulfur metabolism related genes were not involved in their models. Therefore, our Fe&S cycling model provided more detailed insight into the bioleaching process.

Besides, each bioleaching subsystem had its own Fe&S micro-cycles. Because three subsystems had different physicochemical conditions and microbial communities, their Fe&S micro-cycle was different from each other too. For LH and LS, iron oxidation was predominant process in their Fe cycles while iron reduction was more active for Fe cycles in Sediment. Capable of reducing iron, *Acidiphilium* was reported to reduce iron in Tinto River^15^, and it was also the major iron reducers in Sediment of Dexing bioleaching system. Predominant iron-oxidizing bacteria in LH were *Acidithiobacillus*, *Leptospirillum* and *Acidiferrobacter*. Particularly, *Acidiferrobacter* was an acidophilic, thermo-tolerant, facultatively anaerobic iron- and sulfur-oxidizer^35^. It accounted for 15.14% of all reads in LH samples, and thus might play an important role inside the leaching heap where oxygen was scarce. However, H. Korehi *et al*. reported the iron cycles with the involvement of *Acidithiobacillus*, *Sulfobacillus* and *Alicyclobacillus* during pyrite dissolution in mine tailings^14^. It might result from different geochemical conditions of two mining sites^13^,^36^, for pH, oxygen content and energy source were all key factors impacting microbial communities.

Our study showed drastic changes in geochemical properties, as well as significant shift in structure, function and co-occurrence patterns of the microbial communities among LH, LS and Sediment subsystems in Dexing Copper Mine. A biochemical Fe&S cycling model was also constructed to unveil the bioleaching process. Mineral leaching capability of microbial communities would be investigated in the future to relate the shift pattern of microbial communities to bioleaching efficiency directly. As essential elements related to growth of microorganisms, how carbon, nitrogen and phosphorus cycle in bioleaching system also need to be explored further.

## Materials and Methods

### Site description, sampling and 16S rRNA gene sequencing

In this study, a total of 30 samples were collected from LH (1.5 meter deep), LS and Sediment subsystems in the Dexing Copper Mine with 10 replicates for each subsystem. Dexing copper mine is located in Jiangxi Province, China, with longitude ranged from 113°34′36″ to 118°28′58″, and latitude ranged from 24°29′14″ to 30°04′41″. All samples were immediately transferred to the laboratory and stored at −80 °C until DNA extraction or at 4 °C for geochemical property analyses. Microorganisms in LH and Sediment samples were washed by deionized water for several times until there were almost no residual cells, and collected with a 0.22 μm filter paper, while microorganisms in LS were filtered directly. DNA was extracted using a MO BIO PowerSoil DNA Isolation Kit (MO BIO, 12888-100) following the classic protocol^37^. The V4 region of 16S rRNA genes were amplified and sequenced on a MiSeq (Illumina, San Diego, CA). Refer to Supplementary materials for detailed procedures of PCR amplification, purification and library preparation.

### Network construction and characterization

Phylogenetic molecular ecological networks (pMENs) were constructed with 16S rRNA gene sequencing data. As previously described, random matrix theory (RMT)-based approaches were used for network construction with optimized threshold^38^,^39^,^40^. To ensure correlation reliability, OTUs present in at least 5 out of 10 replicates were used for network analysis. The Cytoscape 2.6.0^41^ software was used to visualize the network graphs. Since we are interested in the spatial difference of network interactions, the pMENs were constructed separately for the three subsystems (LH, LS and Sediment). Details about pMENs construction were described in the Supplementary Material.

### S and Fe oxidation and functional gene array

S and Fe oxidation activity was measured to evaluate S and Fe oxidation of microbial communities in the three subsystems. LH and Sediment samples were washed with sterile water immediately after sampling, to wash off the microorganisms in the samples. Then the eluent of LH and Sediment along with LS samples were filtered through a microfiltration membrane (0.22 μm) in order to collect microbial cells. Each collection was cultured in 250 mL flasks containing 100 mL of sterile medium 9 K with the same inoculation concentration (1 × 10^6^ cells/mL), and incubated at 30 °C on a rotary shaker (170 r/min). Initial pH was 2.2. Then S^0^ (10 g/L) and FeSO_4_ (44.7 g/L) were added respectively. The pH value and concentration of Fe^2+^ were measured every 24 hours.

Functional gene array hybridization was conducted to further reveal the functional difference between microbial communities of LH and LS. DNA of 20 samples was extracted and purified as described previously^42^. Amplified DNA was labeled and hybridized with GeoChip 5.0, containing about 60,000 probes in biogeochemical cycling of carbon (C), nitrogen (N), sulfur (S) and phosphorus (P). The hybridized GeoChip data were analyzed as previously described^43^.

## Additional Information

**How to cite this article**: Niu, J. *et al*. The shift of microbial communities and their roles in sulfur and iron cycling in a copper ore bioleaching system. *Sci. Rep.* **6**, 34744; doi: 10.1038/srep34744 (2016).

## Supplementary Material

Supplementary Information

## Figures and Tables

**Table 1:** Physicochemical properties of each sample.

Sample	pH	TON	TOC	DO	Fe^2+^	Fe^3+^	TFe	AVS	CRS	ES	TS	Cu	K	P
LH	3.2	263.1	1243.2	0.73	32.1	36.8	60222	32.7	11354.2	950.7	46746	1288	27112	121.3
LS	2.4	54.1	11.1	1.33	98.7	362.3	500	40.3	34.4	10.1	8227	171	6.32	75.4
Sediment	2.8	1137.3	8362.4	0.39	56.97	90.7	114792	63.6	2764.9	111.9	41496	710	22117	189.6

All values are in ppm, except for pH.

*LH: leaching heap; LS: leaching solution; TON: total organic nitrogen; TOC: total organic carbon; DO: dissolved oxygen; TFe: total Fe; AVS: acid volatile sulfide; CRS: chromium reducible sulfur; ES: elemental sulfur; TS: total sulfur.
