# Supplementary material for: The shift of microbial communities and their roles in sulfur and iron cycling in a copper ore bioleaching system
Source: Sci Rep. 2016 Oct 4;6:34744. doi: 10.1038/srep34744 (PMC5048113; doi:10.1038/srep34744)
Supplement: Supplementary Information [file srep34744-s1.pdf]

The shift of microbial communities and their roles in sulfur and iron cycling in a copper ore bioleaching system (supporting information)

Jiaojiao Niu, Jie Deng, Yunhua Xiao, Zhili He, Xian Zhang, JD Van Nostrand, Yili Liang, Ye Deng, Xueduan Liu, Huaqun Yin

Jiaojiao Niu (jjniu15@hotmail.com)
Jie Deng (jiedeng@ou.edu)
Yunhua Xiao (huazipiaoling.123@163.com)
Zhili He (zhili.he@ou.edu)
Xian Zhang (zixuange2010@126.com)
Joy D. Van Nostrand (Joy.VanNostrand@ou.edu)
Yili Liang (liangyili@hotmail.com)
Ye Deng (yedeng@rcees.ac.cn)
Xueduan Liu (xueduanliu@yahoo.com)
HuaqunYin(yinhuaqun@gmail.com)

Supplementary Tables and Figures

Table S1. Shannon diversity, Pielou evenness and Chao value of microbial communities in each subsystem. Significant differences ($p < 0.05$) among groups are indicated by alphabetic letters.

	Chao value	Shannon diversity	Pielou evenness
LH	571.16	3.01 (a)	0.56 (a)
LS	922.87	1.49 (b)	0.28 (b)
Sediment	780.45	2.74 (a)	0.57 (a)

Table S2. Summary of Fe and S metabolism characteristics of functional bacteria detected in this study.

	Oxidize Fe	Reduce Fe	Oxidize S	Reduce S
Acidithiobacillus	P	P	P	
Leptospirillum	P			
Ferroplasma	P			
Ferrimicrobium	P			
Ferrithrix	P			
Ferritrophicum	P			
Ferrovum	P			
Acidiplasma	P	P		
Thiomonas	P		P	
Sulfobacillus	P	P	P	
Acidiferrobacter	P		P	
Acidiphilium		P		
Comamonas		P		
Aciditerrimonas		P		
Shewanella		P		
Anaeromyxobacter		P		
Metallibacterium		P		
Acidicaldus		P	P	
Thiobacter			P	
Thiobacillus			P	

Metallosphaera			P	
Sulfolobus	p		P	
Sulfurihydrogenibium	P	P	P	P
Geobacter		P		P
Syntrophobacter				P
Desulfitobacterium				P

*P indicates the bacteria could perform the function.[1-24]

Table S3. Normalized intensity of functional gene groups.

Gene category	Subcategory	LH	LS
Sulfur		1649.90±23.02	1662.90±18.71
	sulfur Reduction	**208.60±7.36**	**200.64±3.82**
	sulfide Oxidation	108.27±5.16	110.50±3.76
	sulfite reduction	**761.98±35.11**	**796.53±21.61**
	Sulfur Oxidation	**245.10±10.14**	**234.38±3.71**
Metal Homeostasis		**1485.54±17.68**	**1454.81±8.08**
	Arsenic	590.50±14.85	579.65±7.14
	Chromium	**21.87±2.24**	**19.72±1.005**
	Copper	**17.36±1.12**	**18.76±1.46**
	Mercury	**409.99±12.12**	**389.59±9.12**
Carbon Cycling		**8456.10±93.42**	**8578.20±21.62**
	Carbon degradation	**6080.98±95.07**	**6217.95±33.28**
	Carbon fixation	**2249.88±10.60**	**2224.29±15.32**
Nitrogen		**2553.68±21.95**	**2531.69±13.86**
	Nitrification	**35.93±2.19**	**38.32±1.58**
	Denitrification	**1342.71±29.91**	**1302.60±12.24**
Phosphorus		879.60±12.75	879.43±7.58
	Polyphosphate synthesis	**205.62±6.02**	**210.82±2.07**
	Polyphosphate degradation	**575.01±5.93**	**568.85±3.56**
Organic Remediation		**4537.09±87.17**	**4442.87±39.49**
Secondary metabolism		33.84±1.45	33.08±1.23
Virulence		**327.13±4.45**	**338.14±4.96**
Other		77.12±3.76	78.87±3.04

*Significant differences ($p < 0.05$) are indicated in bold.

Table S4. Topological properties of the empirical pMENs of microbial communities in three groups.

Community	No.of original OTUs	Similarity threshold	Total nodes	Total links	R square of power-law	Average degree (avgK)	Average clustering coefficient (avgCC)	Average path distance (GD)	#module	modularity
LH	183	0.80	154	753	0.702	9.779	0.386	3.315	8	0.417
LS	128	0.80	90	378	0.75	8.4	0.429	3.139	6	0.402
Sediment	68	0.80	53	114	0.775	4.302	0.385	2.983	8	0.564

Table S5. Mantel test of sequencing data with environmental attributes at the genus level.

Genus	pH	DO	Fe^{2+}	Fe^{3+}	TFe	AVS	CRS	ES	TS
Acidiphilium	0.277	0.533	0.931	0.621	0.44	0.494	0.131	0.287	**0.097**
Acidiplasma	0.111	0.963	0.941	0.624	0.921	0.103	**0.069**	0.326	0.567
Aciditerrimonas	0.191	0.578	0.885	0.77	**0.05**	**0.023**	0.802	0.725	0.555
Acidithiobacillus	0.59	**0.004**	**0.004**	0.164	**0.033**	1	0.402	0.478	**0.004**
Comamonas	**0.053**	0.232	0.358	0.985	**0.002**	0.142	0.631	0.704	0.995
Desulfitobacterium	0.102	0.333	0.951	0.797	1	0.124	0.968	0.897	0.723
Ferrimicrobium	**0.064**	1	0.914	0.655	0.97	0.729	**0.001**	**0.001**	0.38
Ferrithrix	0.119	0.914	0.95	0.557	0.808	0.128	**0.048**	0.231	**0.03**
Ferroplasma	**0.045**	0.738	0.906	0.668	0.98	0.266	**0.001**	**0.015**	0.374
Geobacter	0.247	0.953	0.482	0.902	**0.001**	**0.094**	**0.017**	0.153	0.898
Leptospirillum	0.615	**0.007**	0.778	**0.001**	0.14	**0.057**	0.981	0.91	**0.012**
Metallosphaera	1	0.446	0.343	0.799	0.523	0.457	0.934	0.881	0.299
Sulfobacillus	0.758	0.629	0.875	**0.087**	**0.093**	0.751	0.309	0.288	0.726
Sulfolobus	0.296	0.389	0.676	0.768	**0.054**	0.389	0.732	0.563	0.827
Sulfurihydrogenibium	0.133	0.629	0.8	0.555	**0.096**	0.21	0.974	0.984	**0.043**
Thiobacillus	0.975	0.616	0.995	0.664	0.973	0.147	**0.02**	**0.075**	0.114
Thiobacter	0.776	0.595	0.93	0.744	1	**0.031**	**0.004**	**0.074**	0.862
Thiomonas	0.95	0.348	0.899	0.546	0.958	0.462	**0.001**	**0.011**	0.716

Table S6. Mantel test of sulfur metabolizing genes with environmental attributes.

Gene	DO	AVS	CRS	ES	TS	TFe	Fe^{2+}	Fe^{3+}	Cu
aps_aprB	0.57	**0.064**	**0.001**	**0.041**	**0.001**	**0.001**	0.982	0.187	0.242
aps_apra	0.349	**0.044**	**0.001**	**0.029**	**0.001**	**0.001**	0.891	0.391	0.306
sir	0.181	0.114	**0.001**	**0.041**	**0.001**	**0.001**	0.937	0.484	0.287
soxA	0.152	**0.033**	**0.003**	**0.029**	**0.001**	**0.001**	0.746	0.391	0.154
soxB	0.303	**0.051**	**0.001**	**0.036**	**0.001**	**0.001**	0.874	0.216	0.202
soxC	0.447	0.38	**0.002**	0.241	**0.001**	**0.001**	0.285	0.385	0.248
soxV	0.147	**0.009**	**0.002**	**0.052**	**0.002**	**0.001**	0.773	0.336	**0.098**
soxY	0.199	**0.031**	**0.002**	**0.025**	**0.002**	**0.001**	0.94	0.321	0.397
cysI	0.481	**0.04**	**0.001**	**0.041**	**0.001**	**0.001**	0.584	0.326	0.244
cysJ	**0.029**	0.11	**0.001**	**0.029**	**0.001**	**0.001**	0.941	0.394	0.387
dmdA	0.429	**0.04**	**0.002**	**0.039**	**0.001**	**0.001**	0.85	0.353	**0.045**
dsrB	0.183	**0.031**	**0.001**	**0.023**	**0.001**	**0.001**	0.688	0.311	0.364
dsra	0.193	**0.05**	**0.003**	**0.041**	**0.001**	**0.001**	0.913	0.327	0.306
fccab	0.511	**0.037**	**0.001**	**0.029**	**0.001**	**0.001**	0.808	0.308	0.267
sqr	0.296	**0.023**	**0.014**	**0.053**	**0.001**	**0.001**	0.652	0.495	0.53

*Significant impacts ($p < 0.1$) are indicated in bold.
*DO was partial out when analyzing the impact of AVS, CRS, ES, Fe^{2+} and Fe^{3+} on microbial communities.

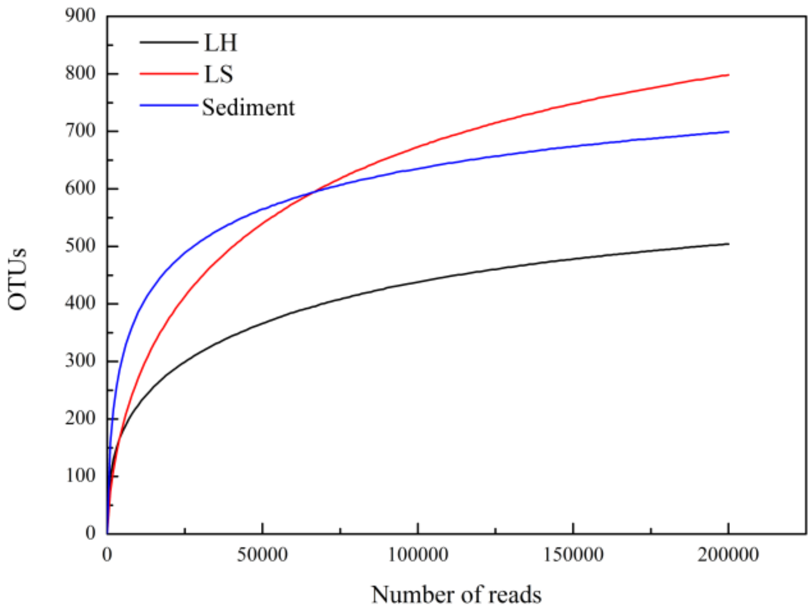

Figure S1. Rarefaction of 16S rRNA gene sequencing in each subsystem.

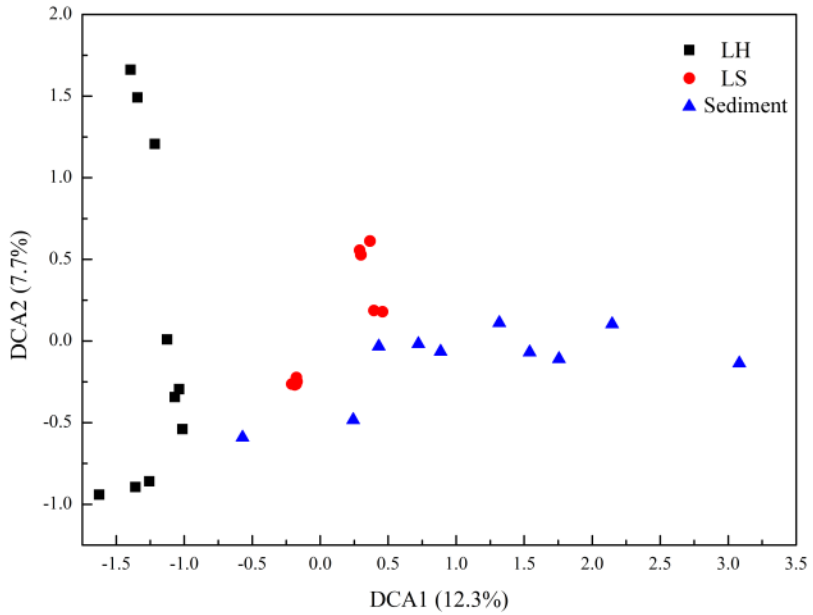

Figure S2. Detrended correspondence analysis (DCA) of 16r RNA gene sequencing data. The values of DCA1 and DCA2 are percentages of total variations that can be attributed to the corresponding axis.

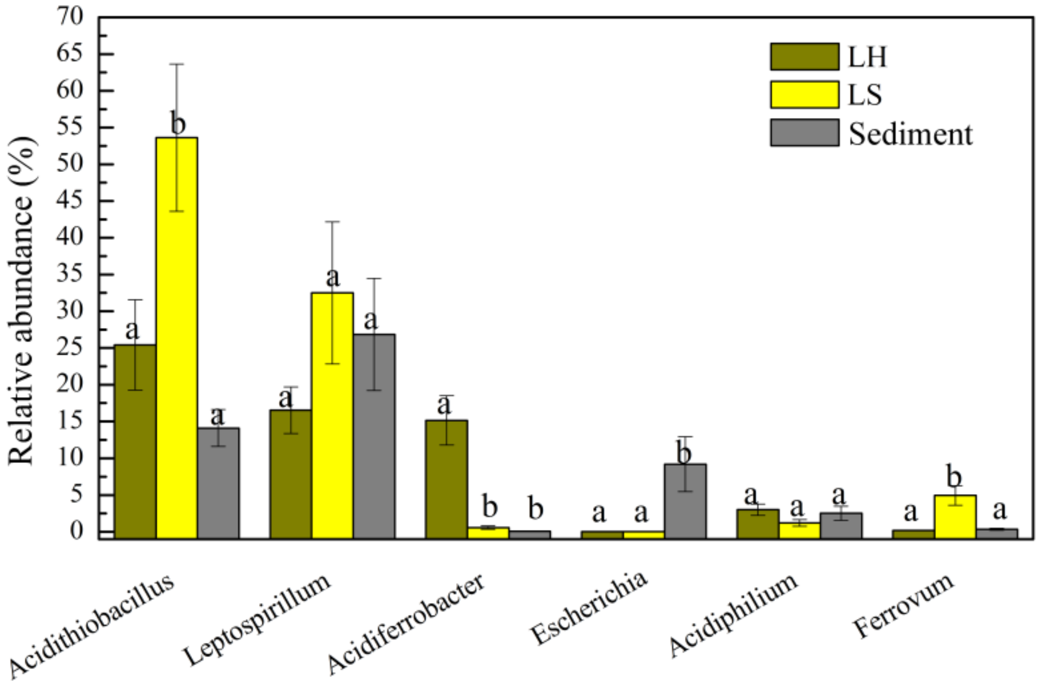

Figure S3. Relative abundances of microbial populations in three groups at the genus level. Significant differences ($p < 0.05$) among groups are indicated by alphabetic letters above the bars.

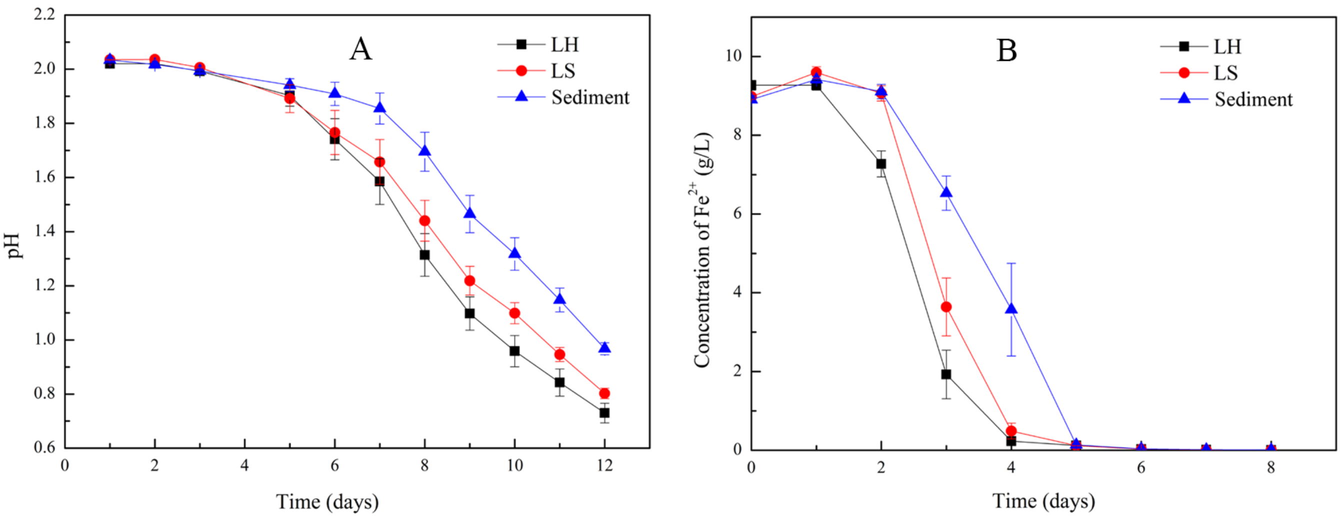

Figure S4. pH (A) and Fe^{2+} concentration (B) changed over time after inoculation of microbial communities.

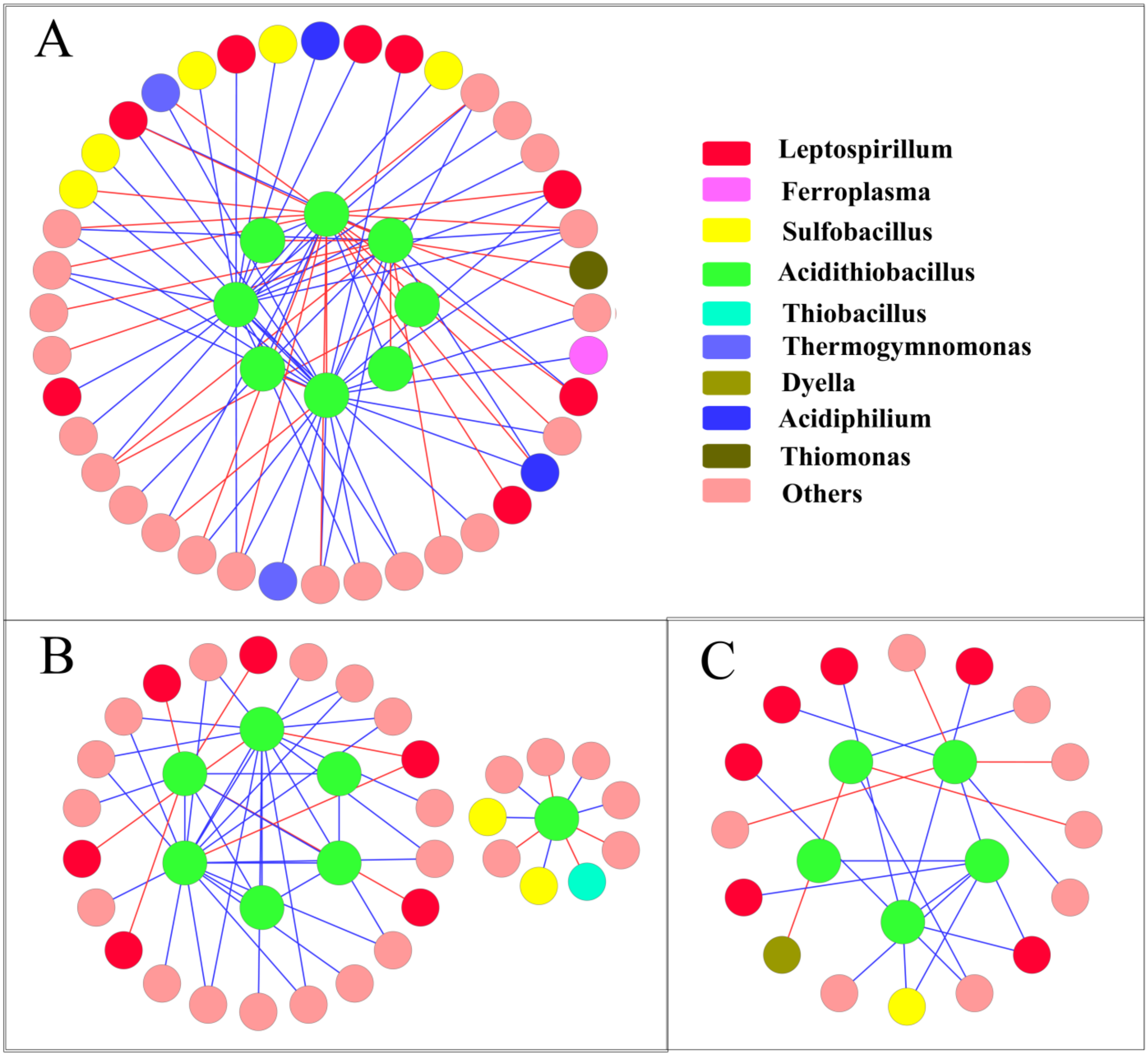

Figure S5. Network interactions of the only OTUs of *Acidithiobacillus* in LS (A) and LH (B) and Sediment (C). Each node signifies an OTU which could correspond to a microbial population. Colors of the nodes indicate different major genera. A blue line indicates a positive interaction between two individual nodes, while a red line indicates a negative interaction.

Supplementary Methods

Geochemical analysis

Elemental composition of samples was analyzed by ICP-AES[25]. pH was measured with a pH meter. The concentrations of Fe^{2+} and Fe^{3+} were measured using phenanthroline spectrophotometry[26]. The samples from LH and sediment were dissolved sufficiently in sterile water (5g/25mL) before pH and Fe assay while LS samples were measured directly. Dissolved oxygen (DO) were measured on-site by use of specific electrodes. Total organic nitrogen (TON) was quantified by Kjeldahl distillation[27]. The amount of total organic carbon (TOC) was analyzed by potassium dichromate oxidation-ferrous sulphate titrimetry[28]. Acid volatile sulfide (AVS), chromium reducible sulfur (CRS) and elemental sulfur (ES) were measured with iodometry[29].

DNA amplification, Illumina sequencing and data processing

The V4 region of the 16S rRNA genes was amplified with the primer pair 515F (50-GTGCCAGCMGCCGCGGTAA-30) and 806R (50-GGACTACHVGGGTWTCTAAT-30) combined with Illumina adapter sequences, a pad and a linker of two bases, as well as barcodes on the reverse primers[30]. Sample libraries were generated from purified PCR products. The Miseq 500 cycles kit was used for 2×250 bp paired-ends sequencing on Miseq machine (Illumina, San Diego, CA, USA). Raw sequences with perfect matches to barcodes were split to sample libraries and were trimmed using BTRIM with threshold of QC higher than 20 over 5 bp window size and the minimum length of 100 bp[31]. Forward and reverse reads with at least 10 bp overlap and lower than 5% mismatches were joined using FLASH[32]. After trimming of ambiguous bases (i.e. N), joined sequences with lengths between 240 and 260 bp were subjected to chimera removal by U-Chime[33]. OTU clustering was through UCLUST at 97% similarity level[34], and taxonomic assignment was through RDP classifier[35] with a minimal 50%

confidence estimate. The above steps were performed through the Galaxy pipeline (http://zhoulab5.rccc.ou.edu/) developed by Y. Qin (unpublished). Singletons were removed for downstream analyses. Samples were rarefied at 20,000 sequences per sample. All the 16S rRNA sequences were deposited in GenBank database with the accession numbers of KT067912 - KT069216.

Bacterial community diversity was calculated using Shannon-Weiner's diversity (H′) and Pielou evenness. Dissimilarity tests were based on the Bray-Curtis dissimilarity using analysis of similarities (ANOSIM)[36]. Differences in abundances across sites were determined by analysis of variance (ANOVA) using Least Significant Difference (LSD) test[37]. Multivariate statistical analyses of sequencing data were conducted, including detrended correspondence analysis (DCA) for comparing the different microbial communities. The Mantel test[38] was used to calculate the correlations between bacterial abundance and environmental attributes. All the analyses were performed in R[39] with the packages vegan v. 1.11-3 or online (http://ieg.ou.edu/).

Phylogenetic molecular network construction

As previously described, random matrix theory (RMT)-based approaches were used for network construction, hub and connector gene identification, and topological property determination with an automatic threshold[40,41]. To ensure correlation reliability, OTUs in at least 5 out of 10 replicates were used for network analysis. Various network properties such as average degree, average path distance, average clustering coefficient and modularity index were calculated and the network modules were generated using rapid greedy modularity optimization[42].

The phylogenetic molecular ecological networks (pMENs) were constructed with 16S rRNA gene sequencing data. First, a Pearson correlation matrix was constructed[43]. The correlation matrix was then converted to a similarity matrix, which measures the degree of concordance between the abundance

profiles of OTUs across different samples by taking the absolute values of the correlation matrix[43]. Subsequently, an adjacency matrix, which encodes the connection strength between each pair of nodes, was derived from the similarity matrix by applying an appropriate threshold, which was defined using the RMT-based network approach as previously described[41,44]. Finally, the Cytoscape 2.6.0[45] software was used to visualize the network graphs. Other information about genes (e.g., taxonomy, relative abundance) and edge information (e.g., weights and positive and negative correlations) was also imported into the software and visualized in the network figures. Since we are interested in the spatial difference of network interactions, the pMENs were constructed separately for three subsystems (LH, LS and SD).

Microarray hybridization and data processing

GeoChip 5.0 was manufactured by Agilent (Agilent Technologies Inc., Santa Clara, CA) in the 8 × 60 K format. 600 ng of purified soil DNA of each sample was labeled with the fluorescent dye Cy-3 (GE Healthcare, CA, USA) using a random priming method as described previously[46] purified using a QIAquick Purification kit (Qiagen, CA, USA), and dried in a SpeedVac (Thermo Savant, NY, USA) into a powder. Subsequently, the labeled DNA was resuspended into 27.5 μl of DNase/RNase-free distilled water, and mixed completely with 42 μl of hybridization solution, containing 1×Acgh blocking, 1×HI-RPM hybridization buffer, 10 pM universal standard DNA, 0.05 μg/μl Cot-1 DNA, and 10% formamide (final concentrations). After these, the solution was denatured at 95 °C for 3 min, and then incubated at 37 °C for 30 min, then hybridized with GeoChip 5.0 arrays (60 K). GeoChip hybridization was preceded at 67 °C in Agilent hybridization oven for 24 h. After hybridization, the slides were washed using Agilent Wash Buffers at room temperature. Then the arrays were scanned at 633 nm by a laser power of 100% and a photomultiplier tube gain of 75% with a NimbleGen MS200 Microarray

Scanner (Roche NimbleGen, Inc., Madison, WI, USA). The images data were extracted by following Agilent Feature Extraction program.

The microarray data were preprocessed by the microarray analysis pipeline on IEG website (http://ieg.ou.edu/microarray/) as previously described[46]. The main steps were in the following steps: (i) removing the spots of poor quality, which was a signal to noise ratio of less than 2.0; (ii) the relative abundance of each soil sample was calculated by dividing the total intensity of the detected probes, then multiplying a constant and taking the natural logarithm transformation; (iii) the detected probes in only two out of eight samples from the same sampling sites were removed.

References

1 Aguiar, P., Beveridge, T. & Reysenbach, A.-L. Sulfurihydrogenibium azorense, sp. nov., a thermophilic hydrogen-oxidizing microaerophile from terrestrial hot springs in the Azores. *International Journal of Systematic and Evolutionary Microbiology* **54**, 33-39 (2004).

2 Fredrickson, J. K. & Gorby, Y. A. Environmental processes mediated by iron-reducing bacteria. *Current opinion in biotechnology* **7**, 287-294 (1996).

3 Valdes, J., Ossandon, F., Quatrini, R., Dopson, M. & Holmes, D. S. Draft genome sequence of the extremely acidophilic biomining bacterium Acidithiobacillus thiooxidans ATCC 19377 provides insights into the evolution of the Acidithiobacillus genus. *Journal of bacteriology* **193**, 7003-7004 (2011).

4 Coram, N. J. & Rawlings, D. E. Molecular relationship between two groups of the genus Leptospirillum and the finding that Leptospirillum ferriphilum sp. nov. dominates South African commercial biooxidation tanks that operate at 40 C. *Applied and environmental microbiology* **68**, 838-845 (2002).

5 Golyshina, O. V. *et al.* Ferroplasma acidiphilum gen. nov., sp. nov., an acidophilic, autotrophic, ferrous-iron-oxidizing, cell-wall-lacking, mesophilic member of the Ferroplasmaceae fam. nov., comprising a distinct lineage of the Archaea. *International Journal of Systematic and Evolutionary Microbiology* **50**, 997-1006 (2000).

6 Johnson, D. B., Bacelar-Nicolau, P., Okibe, N., Thomas, A. & Hallberg, K. B. Ferrimicrobium acidiphilum gen. nov., sp. nov. and Ferrithrix thermotolerans gen. nov., sp. nov.: heterotrophic, iron-oxidizing, extremely acidophilic actinobacteria. *International Journal of Systematic and Evolutionary Microbiology* **59**, 1082-1089 (2009).

7 Weiss, J. V. *et al.* Characterization of neutrophilic Fe (II)-oxidizing bacteria isolated from the rhizosphere of wetland plants and description of Ferritrophicum radicicola gen. nov. sp. nov., and Sideroxydans paludicola sp. nov. *Geomicrobiology Journal* **24**, 559-570 (2007).

8 Johnson, D. B., Hallberg, K. B. & Hedrich, S. Uncovering a microbial enigma: isolation and characterization of the streamer-generating, iron-oxidizing, acidophilic bacterium “Ferrovum myxofaciens”. *Applied and environmental microbiology* **80**, 672-680 (2014).

9 Golyshina, O. V. *et al.* Acidiplasma aeolicum gen. nov., sp. nov., a euryarchaeon of the family Ferroplasmaceae isolated from a hydrothermal pool, and transfer of Ferroplasma cupricumulans to Acidiplasma cupricumulans comb. nov. *International Journal of Systematic and Evolutionary Microbiology* **59**, 2815-2823 (2009).

10 Moreira, D. & Amils, R. Phylogeny of Thiobacillus cuprinus and other mixotrophic thiobacilli: proposal for Thiomonas gen. nov. *International Journal of Systematic and Evolutionary Microbiology* **47**, 522-528 (1997).

11 Clark, D. A. & Norris, P. R. Acidimicrobium ferrooxidans gen. nov., sp. nov.: mixed-culture ferrous iron oxidation with Sulfobacillus species. *Microbiology* **142**, 785-790 (1996).

12 Hallberg, K. B., Hedrich, S. & Johnson, D. B. Acidiferrobacter thiooxydans, gen. nov. sp. nov.; an acidophilic, thermo-tolerant, facultatively anaerobic iron-and sulfur-oxidizer of the family Ectothiorhodospiraceae. *Extremophiles : life under extreme conditions* **15**, 271-279 (2011).

13 Küsel, K., Dorsch, T., Acker, G. & Stackebrandt, E. Microbial reduction of Fe (III) in acidic sediments: isolation of Acidiphilium cryptum JF-5 capable of coupling the reduction of Fe (III) to the oxidation of glucose. *Applied and environmental microbiology* **65**, 3633-3640 (1999).

14 Wu, C.-Y., Zhuang, L., Zhou, S.-G., Li, F.-B. & Li, X.-M. Fe (III)-enhanced anaerobic transformation of 2, 4-dichlorophenoxyacetic acid by an iron-reducing bacterium Comamonas koreensis CY01. *FEMS microbiology ecology* **71**, 106-113 (2010).

15 Itoh, T., Yamanoi, K., Kudo, T., Ohkuma, M. & Takashina, T. Aciditerrimonas ferrireducens gen. nov., sp. nov., an iron-reducing thermoacidophilic actinobacterium isolated from a solfataric field. *International Journal of Systematic and Evolutionary Microbiology* **61**, 1281-1285 (2011).

16 Kim, B. H., Kim, H. J., Hyun, M. S. & Park, D. H. Direct electrode reaction of Fe (III)-reducing bacterium, Shewanella putrefaciens. *Journal of Microbiology and Biotechnology* **9**, 127-131 (1999).

17 Sanford, R. A., Cole, J. R. & Tiedje, J. M. Characterization and description of Anaeromyxobacter dehalogenans gen. nov., sp. nov., an aryl-halorespiring facultative anaerobic myxobacterium. *Applied and environmental microbiology* **68**, 893-900 (2002).

18 Ziegler, S. *et al.* Metallibacterium scheffleri gen. nov., sp. nov., an alkalinizing gammaproteobacterium isolated from an acidic biofilm. *International Journal of Systematic and Evolutionary Microbiology* **63**, 1499-1504 (2013).

19 Johnson, D. B., Stallwood, B., Kimura, S. & Hallberg, K. B. Isolation and characterization of Acidicaldus organivorus, gen. nov., sp. nov.: a novel sulfur-oxidizing, ferric iron-reducing thermo-acidophilic heterotrophic Proteobacterium. *Arch. Microbiol.* **185**, 212-221 (2006).

20 Hirayama, H., Takai, K., Inagaki, F., Nealson, K. H. & Horikoshi, K. Thiobacter subterraneus gen. nov., sp. nov., an obligately chemolithoautotrophic, thermophilic, sulfur-oxidizing bacterium from a subsurface hot aquifer. *International Journal of Systematic and Evolutionary Microbiology* **55**, 467-472 (2005).

21 Kelly, D. P. & Wood, A. P. Reclassification of some species of Thiobacillus to the newly designated genera Acidithiobacillus gen. nov., Halothiobacillus gen. nov. and Thermithiobacillus gen. nov. *International Journal of Systematic and Evolutionary Microbiology* **50**, 511-516 (2000).

22 Huber, G., Spinnler, C., Gambacorta, A. & Stetter, K. O. Metallosphaera sedula gen, and sp. nov. represents a new genus of aerobic, metal-mobilizing, thermoacidophilic archaebacteria. *Systematic and applied microbiology* **12**, 38-47 (1989).

23 Brock, T. D., Brock, K. M., Belly, R. T. & Weiss, R. L. Sulfolobus: a new genus of sulfur-oxidizing bacteria living at low pH and high temperature. *Archiv für Mikrobiologie* **84**, 54-68 (1972).
24 Druschel, G. K., Baker, B. J., Gihring, T. M. & Banfield, J. F. Acid mine drainage biogeochemistry at Iron Mountain, California. *Geochemical Transactions* **5**, 13-32 (2004).
25 Ramsey, M. H. & Thompson, M. High-accuracy analysis by inductively coupled plasma atomic emission spectrometry using the parameter-related internal standard method. *Journal of Analytical Atomic Spectrometry* **2**, 497-502 (1987).
26 Stookey, L. L. Ferrozine---a new spectrophotometric reagent for iron. *Analytical chemistry* **42**, 779-781 (1970).
27 McKenzie, H. A. & Wallace, H. S. The Kjeldahl determination of Nitrogen: A critical study of digestion conditions-Temperature, Catalyst, and Oxidizing agent. *Australian Journal of Chemistry* **7**, 55-70 (1954).
28 Liu, C., Zhu, Z., He, X., Zhang, B. & Xia, N. Rapid determination of organic carbon in marine sediment samples by potassium dichromate oxidation-ferrous sulphate titrimetry. *Rock Miner Anal* **6**, 205–208 (2007).
29 Bremanis, E., Deering, J., Meade, C. & KEYWORTH. Vol. 7 459-& (MATERIALS RESEARCH SOCIETY 506 KEYSTONE DR, WARRENDALE, PA 15086, 1967).
30 Caporaso, J. G. *et al.* Ultra-high-throughput microbial community analysis on the Illumina HiSeq and MiSeq platforms. *The ISME journal* **6**, 1621-1624, doi:10.1038/ismej.2012.8 (2012).
31 Kong, Y. Btrim: A fast, lightweight adapter and quality trimming program for next-generation sequencing technologies. *Genomics* **98**, 152-153, doi:http://dx.doi.org/10.1016/j.ygeno.2011.05.009 (2011).
32 Magoč, T. & Salzberg, S. L. FLASH: fast length adjustment of short reads to improve genome assemblies. *Bioinformatics* **27**, 2957-2963 (2011).
33 Edgar, R. C., Haas, B. J., Clemente, J. C., Quince, C. & Knight, R. UCHIME improves sensitivity and speed of chimera detection. *Bioinformatics* **27**, 2194-2200 (2011).
34 Edgar, R. C. Search and clustering orders of magnitude faster than BLAST. *Bioinformatics* **26**, 2460-2461, doi:10.1093/bioinformatics/btq461 (2010).
35 Wang, Q., Garrity, G. M., Tiedje, J. M. & Cole, J. R. Naive Bayesian classifier for rapid assignment of rRNA sequences into the new bacterial taxonomy. *Applied and environmental microbiology* **73**, 5261-5267, doi:10.1128/AEM.00062-07 (2007).
36 CLARKE, K. R. Non-parametric multivariate analyses of changes in community structure. *Australian Journal of Ecology* **18**, 117-143 (1993).
37 Calinski. PRINCIPLES AND PROCEDURES OF STATISTICS - A BIOMETRICAL APPROACH, 2ND EDITION - STEEL, RGD, TORRIE,JH. *Biometrics* **37**, 859-860 (1981).
38 E., S. P., C., L. J. & R., S. R. Multiple regression and correlation extensions of the Mantel test of matrix correspondence. *Syst zool* **35**, 627-632 (1986).
39 Team, R. C. (ISBN 3-900051-07-0, 2014).
40 Zhou, J. *et al.* Functional Molecular Ecological Networks. *mBio* **1**, e00169-00110-e00169-00119, doi:10.1128/mBio.00169-10 (2010).
41 Luo, F. *et al.* Constructing gene co-expression networks and predicting functions of unknown genes by random matrix theory. *BMC bioinformatics* **8**, 299, doi:10.1186/1471-2105-8-299 (2007).
42 Deng, Y. *et al.* Molecular ecological network analyses. *BMC bioinformatics* **13**, 113 (2012).
43 Horvath, S. & Dong, J. Geometric interpretation of gene coexpression network analysis. *PLoS*

Comput. Biol **4(8)**, e1000117, doi:10.1371/ (2008).
44 Luo, F., Zhong, J. X., Yang, Y. F., Scheuermann, R. H. & Zhou, a. J. Z. Application of random matrix theory to biological networks. *Phys. Lett. A* **357**, 420 – 423 (2006).
45 Cline, M. S. *et al.* Integration of biological networks and gene expression data using cytoscape. *Nat. Protoc* **2**, 2366 –2382, doi:10.1038/nprot.2007.324 (2007).
46 Tu, Q. *et al.* GeoChip 4: a functional gene ‐ array ‐ based high ‐ throughput environmental technology for microbial community analysis. *Molecular ecology resources* **14**, 914-928 (2014).
